# Modulation of *CYP19* expression by cabbage juices and their active components: indole-3-carbinol and 3,3′-diindolylmethene in human breast epithelial cell lines

**DOI:** 10.1007/s00394-012-0455-9

**Published:** 2012-10-23

**Authors:** Barbara E. Licznerska, Hanna Szaefer, Marek Murias, Agnieszka Bartoszek, Wanda Baer-Dubowska

**Affiliations:** 1 Department of Pharmaceutical Biochemistry, Poznan University of Medical Sciences, Poznan, Poland; 2 Department of Toxicology, Poznan University of Medical Sciences, Poznan, Poland; 3 Department of Food Chemistry, Technology and Biotechnology, Gdansk University of Technology, Gdansk, Poland

**Keywords:** CYP19, Cabbage juices, Indole-3-carbinol, Diindolylmethane, Apoptosis, Breast cancer chemoprevention

## Abstract

**Purpose:**

The aim of this study was to evaluate the effect of white cabbage and sauerkraut juices of different origin and indole-3-carbinol (I3C) and diindolylmethane (DIM) on expression of *CYP19* gene encoding aromatase, the key enzyme of estrogen synthesis.

**Methods:**

Human breast cell lines (MCF7, MDA-MB-231 and MCF10A) were examined to compare the action of cabbage juices versus their active components (I3C, DIM). Real-time PCR and Western blot were used in order to analyse *CYP19* mRNA and protein, respectively.

**Results:**

Remarkable differences in the effect on *CYP19* transcript and protein level were found between the cabbage juices (in 2.5–25 mL/L concentrations) and indoles (in 2.5–50 μM doses) in the three cell lines. While cabbage juices at the lower doses diminished the aromatase expression in nontumorigenic/immortalized MCF10A breast cells (0.25–0.86-fold change, *P* < *0.05*), I3C and DIM were more efficient in decreasing the aromatase expression in estrogen-dependant MCF7 breast cancer cells (0.24–0.82-fold change, *P* < *0.05*). Inhibition of aromatase by juice obtained from cabbage grown on industrial farm was correlated with the induction of apoptosis (1.7–1.8-fold change, *P* < *0.01*) in MCF10A cells. In estrogen-independent MDA-MB-231 cells, up-regulation of *CYP19* expression by I3C and DIM (1.5–2.0-fold change, *P* < *0.05*) was observed. Similarly, in MCF7 cells juices increased aromatase expression (1.1–2.2-fold change, *P* < *0.05*).

**Conclusion:**

These results, particularly that obtained in nontumorigenic/immortalized MCF10A cells, suggest that chemopreventive activity of cabbage against breast cancer observed in epidemiological studies may be partly explained by inhibition of the aromatase expression.

## Introduction

Breast cancer is the most common cancer of women and the leading cause of cancer mortality among women worldwide. Improvements in survival in the industrial world seen in recent decades have been attributed not only to early detection and treatment improvement, but also prophylaxis [1]. Estrogens play an important role in breast cancer development acting as promoters and initiators of carcinogenesis process. The latter activity is related to mutations caused by certain estrogen metabolites [2, 3]. Thus, estrogens are classified as carcinogenic in humans and one of the most important risk factor of breast cancer [4]. Aromatase, a cytochrome P450 encoded by *CYP19*, is the enzyme that synthesizes estrogens by converting C19 androgens into aromatic C18 estrogenic steroids. Several studies have shown that there is a high expression of aromatase in breast cancer tissue [5]. When compared to circulating estrogen, in situ produced estrogen has also been shown to play significant role in breast cancer growth [6]. Therefore, suppression of in situ estrogen formation, particularly in the breast of postmenopausal women by aromatase inhibitors (AIs), is considered as a useful way to prevent and treat breast cancer in these patients.

Many epidemiological studies have shown that a diet high in fruits and vegetables can reduce breast cancer incidence [7]. Moreover, it was demonstrated that many phytochemicals are aromatase inhibitors suppressing in situ estrogen biosynthesis [8, 9]. Epidemiological studies have also demonstrated that consumption of *Brassica* vegetables is associated with a lower incidence of cancers [10]. In Central and Eastern European diet the most common *Brassica* genus is white cabbage and its fermented product, sauerkraut. However, in contrast to the other *Cruciferae* representatives, anti-carcinogenic activity of white cabbage and sauerkraut were less extensively studied. Epidemiological migrant studies have shown that consumption of these food items during adolescence was associated with a 72 % reduced risk of breast cancer [11]. Like other vegetables, *Brassica* contain a number of phytochemicals with chemopreventive properties. However, they are unique in that they are rich sources of glucosinolates (GLS). Depending on the type and conditions of processing, GLS undergo either enzymatic hydrolysis or thermal degradation resulting in the formation of biologically active compounds including indoles (e.g., indole-3-carbinol, I3C and 3,3′-diindolylmethane, DIM). The effect of fermentation on GLS degradation products has been poorly recognized [12, 13]. The formation of some specific products like ascorbigen (ABG) is suggested [14]. I3C and its condensation product, DIM, stimulates a number of cellular responses that are proapoptotic, anti-proliferative, and anti-estrogenic [15–17]. Besides, all the compounds mentioned above may potentially affect the estrogen biosynthesis pathways.

The aim of this study was to evaluate the effect of raw cabbage and sauerkraut juices of different origins (industrial or organic farming) and their major indole components (I3C and DIM) on aromatase expression in two breast cancer cell lines differing in estrogen receptor status (MCF7—estrogen dependant and MDA-MB-231—estrogen independent) and comparison with nontumorigenic/immortalized breast cell line (MCF10A).

## Methods and materials

### Materials

Indole-3-carbinol, dithiothreitol, antibiotics solution (10^4^U penicillin, 10 mg streptomycin, 25 μg amphotericin B), bovine serum albumin, dimethylsulfoxide (DMSO), fetal bovine serum, Dulbecco’s Modified Eagle’s Medium (DMEM), hydrocortisone, 10 mg/ml insulin, 100 μg/ml EGF, horse serum, 3-(4,5-dimethylthiazol-2-yl)-2,5-diphenyltetrazolium bromide (MTT), RIPA buffer, trypsin, Tris, tRNA from *Escherichia coli* were purchased from Sigma Chemicals Co. (St Louis, MO, USA). 3,3′-diindolylmethane was obtained from LKT Laboratories (St. Paul, MN, USA). Antibodies were supplied by Santa Cruz Biotechnology (Santa Cruz, CA, USA). Rainbow colored protein molecular weight marker was purchased from Amersham Pharmacia Biotechnology (Piscataway, NJ, USA). Protease inhibitor tablets were obtained from Roche Diagnostics GmbH (Penzberg, Germany). All other chemicals were commercial products of the highest purity available. I3C and DIM were dissolved in DMSO at a concentration of 100 mM and stored at −20 °C.

### Preparation of juices

The following juices were used in the experiments: SI, sauerkraut juice from industrial farming; RI, raw juice from industrial farming; SO, sauerkraut juice from organic farming; RO, raw juice from organic farming. Fresh white cabbage was purchased in a wholesale shop supplying the area of Gdansk (Poland) in vegetables and from organic farm “FOHAT” (certificate N°92042A). The juices’ preparation and standardization was performed as previously described [18, 19]. In fresh juice, the average content of GLS was 3.283–4.623 μmol/g of dry mass and of I3C was 11.37–14.81 μM. In sauerkraut juice, the content of most GLS was below the level of detection and I3C was 20.53–32.10 μM. Before adding to the cell culture medium, sauerkraut juices were neutralized with NaOH to pH ~ 7.4 to avoid acidifying cell culture medium and sterilized by filtration through Ø22 μm filters.

### Cell culture and treatment

MCF7 (ECACC 86012803) and MDA-MB-231 (ECACC 92020424) cells were purchased from the European Collection of Cell Cultures (Salisbury, Wiltshire, UK). MCF10A (ATTC CRL-10317) cell line was a gift of Dr Blazej Rubis (Dept Clinical Chem Molecular Diagnostics, PUMS). MCF7 and MDA-MB-231 cells were cultured in DMEM supplemented with 10 % fetal bovine serum and 1 % antibiotics solution. To assess the effects of tested compounds, the cells were grown in the presence of 5 % fetal bovine serum. MCF10A cells were cultured in DMEM supplemented with 0.1 % insulin solution, 0.02 % EGF solution, 0.05 % hydrocortisone solution, 5 % horse serum, and 1 % antibiotics solution. All cell lines were routinely maintained in T75 flasks in a 37 °C humidified environment of 5 % CO_2_/95 % air and were passed twice a week using 0.05 % trypsin/0.02 % EDTA. Experiments were conducted at a cell density of 70 % confluency. After 24 h preincubation, the cells were treated with raw cabbage, sauerkraut juices, I3C or DIM at the doses selected based on cytotoxicity assay: 2.5–25 mL/L of juices; 10–50 μM of I3C, and 2.5–10 μM of DIM. The incubation was continued for subsequent 72 h. Control cells were treated with vehicle (DMSO or water). The concentration of DMSO did not exceed 0.1 %.

### Cytotoxicity assay

Cytotoxicity was measured using the MTT assay according to standard protocols. The cells were seeded in a 96-well culture plate. After 24 h preincubation in the culture medium, 2.5–100 mL/L of cabbage juices or 5–100 μM of indoles (I3C and DIM) were added and the cells were incubated for 72 h. Subsequently, the cells were washed with PBS buffer, and fresh medium containing MTT salt (0.5 mg/mL) was added to the wells. After 4 h incubation, the formazan crystals were dissolved in acidic isopropanol and the absorbance was measured at 570 and 690 nm.

### Apoptosis detection

The cell apoptosis ELISA detection kit (Roche, Palo Alto, CA, USA) was used to detect apoptosis in breast cancer cells treated for 72 h with cabbage juices, I3C or DIM according to manufacturer’s protocol. Briefly, 10^4^ of each cell line cells were used in a 48-well culture plate, and the cytoplasmic histone/DNA fragments were extracted and bound to immobilized anti-histone antibody. Subsequently, the peroxidase-conjugated anti-DNA antibody was used for the detection of immobilized histone/DNA fragments. After addition of peroxidase substrate, the absorbance was measured at 405 and 490 nm.

### Measurement of aromatase mRNA transcript (real-time PCR)

Total RNA was isolated using the GenElute Mammalian Total RNA Miniprep Kit (Sigma, St Louis, MO, USA) according to manufacturer’s recommendations and subjected to reverse transcription using the RevertAid First Strand cDNA Synthesis Kit (Fermantas, St. Leon-Rot, Germany), followed by quantitative real-time PCR. For real-time analyses, the Maxima SYBR Green/ROX qPCR Master Mix (Fermentas) and BioRad Chromo4 were used. The protocol started with a 5 min enzyme activation at 95 °C, followed by 40 cycles of 95 °C for 15 s; 56 °C for 20 s; 72 °C for 40 s and the final elongation at 72 °C for 5 min. The melting curve analysis was used for product size verification. Experiments were normalized for the expression of *TBP* and *PBGD*. The Pfaffl relative method was used for fold change quantification. Following primers were used: forward and reverse—*CYP19* (MCF7 and MCF10A: 5′CAAGAAGAGCGTGTTAGAGG3′ and 5′CTGGACAGGTTGGAGGAG3′; MDA-MB-231: 5′GACAGGTTGGAGGAGGTG3′ and 5′CAAGAAGAGCGTGTTAGAGG3′); *PBGD* (5′TCAGATAGCATACAAGAGACC3′ and 5′TGGAATGTTACGAGCAGTG3′); *TBP* (5′GGCACCACTCCACTGTATC3′ and 5′GGGATTATATTCGGCGTTTCG3′).

### Measurement of aromatase protein level (Western blot)

The adherent and floating cells were harvested and lysed in RIPA buffer supplemented with proteinase inhibitors and incubated on ice for 60 min. Cell lysate was centrifuged at 14,000 rpm for 15 min, and the supernatant was recovered. Equal amounts of protein (100 μg) were subjected to 10 % SDS-PAGE slab gels and transferred to nitrocellulose membranes (Immobilon-P; Millipore, Bedford, MA, USA). After blocking with 10 % skimmed milk, the proteins were probed with polyclonal goat CYP19 and polyclonal rabbit β-actin antibodies (Santa Cruz, CA, USA). As the secondary antibodies, the alkaline phosphatase-labeled anti-goat IgG or anti-rabbit IgG were used. Protein contents were measured using albumin as a standard and the β-actin protein as an internal control. The amount of immunoreactive product in each lane was determined by densitometric scanning using a BioRad GS710 Image Densitometer (BioRad Laboratories, Hercules, CA, USA). The values were calculated as relative absorbance units (RQ) per mg protein.

### Statistical analysis

Statistical analysis was performed by one-way ANOVA. The statistical significance between the experimental groups and their respective controls was assessed by Tukey’s post hoc test at *P* < 0.05.

## Results

### Effect of cabbage juices and indoles on cytotoxicity

**Fig. 1:**
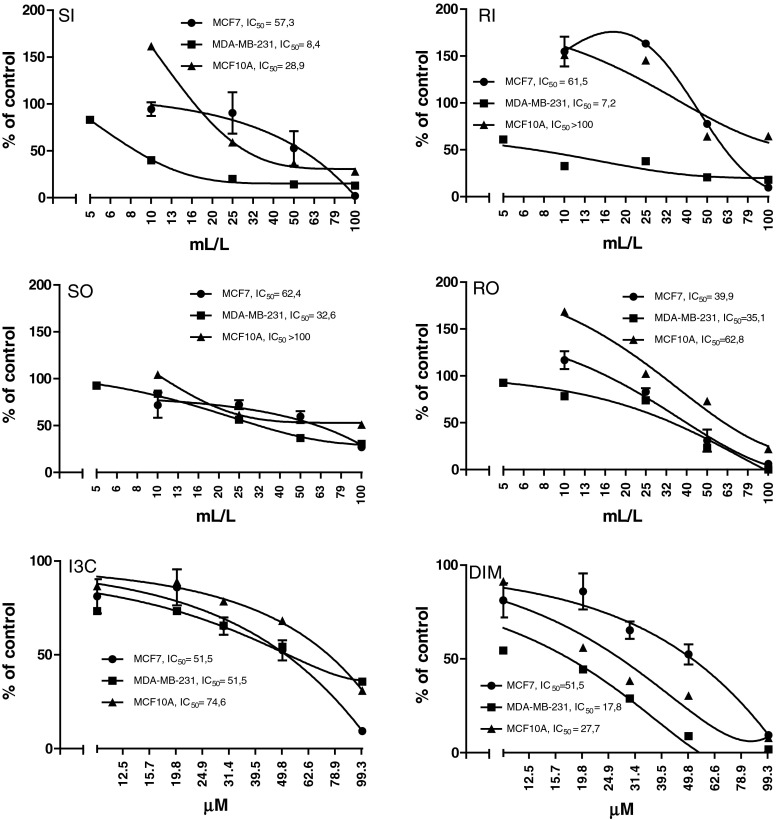
The effect of cabbage juices and indoles on the cytotoxicity of MCF7, MDA-MB-231, and MCF10A cells. Mean values from three separate experiments run in triplicate ±SEM are shown

Figure 1 presents the data on cytotoxicity. Cabbage and sauerkraut juices at the concentrations above 25 mL/L significantly reduced the cytotoxicity, particularly MDA-MB-231 cells (IC_50_: 7.2–35.1). DIM was more toxic than I3C (IC_50_: 17.8–51.5 and IC_50_: 51.5–74.6, respectively) and similarly as in case of cabbage juices the MDA-MB-231 cells were the most sensitive (IC_50_: 17.8–51.5). In subsequent experiments, the nontoxic concentrations (cytotoxicity above 70 % based on MTT assay) were used.

### Effect of cabbage juices and indoles on the expression of CYP19

The expression of *CYP19* was determined at the transcript and protein levels, and no significant differences in the constitutive expression were found between the cell lines tested. However, treatment with cabbage juices or indoles changed *CYP19* transcript and protein levels in a dose and cell line-dependent manner.

**Fig. 2:**
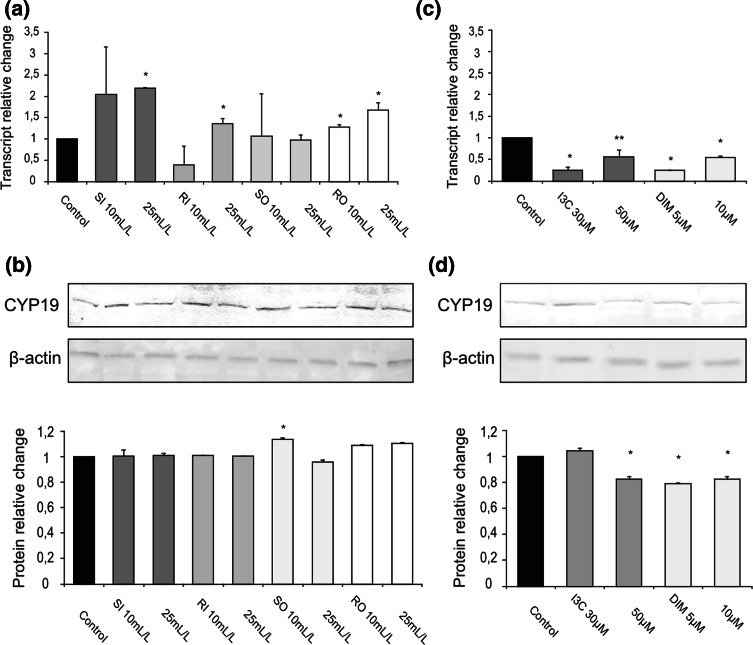
The effect of 72 h incubation with cabbage juices (**a**, **b**) and indoles (**c**, **d**) on the level of the *CYP19* transcript (**a**, **c**) and protein (**b**, **d**) in MCF7 cells. The values were calculated as a relative change in transcript or protein level in comparison with control cells (expression equals 1). The mean values ± SEM from three independent experiments run in duplicate (protein) or triplicate (mRNA) are presented. The *single* *asterisk above the bar* denotes significant difference of mean values from the control group (*P* < 0.05); *double asterisk* indicates trend toward significant difference of mean values from the control group (*P* < 0.054)

In MCF7 cells, both raw cabbage (except 10 mL/L of RI) and sauerkraut juices increased the *CYP19* transcript (1.2–2.0-fold change, Fig. 2a) in comparison with control cells (treated with vehicle only). The higher increase was observed in the case of sauerkraut juice. However, treatment with raw cabbage and sauerkraut juices had no effect on protein level (except 10 mL/L of SO, Fig. 2b). In contrast to cabbage juices, DIM (doses 5 and 10 μM) significantly reduced both *CYP19* transcript (0.24–0.54-fold change, Fig. 2c) and protein levels (0.79–0.82-fold change, Fig. 2d). Also treatment with I3C resulted in evident decrease in mRNA level (0.25–0.56-fold change, doses 30 and 50 μM, Fig. 2c) and protein level (0.82-fold change, dose 50 μM, Fig. 2d).

**Fig. 3:**
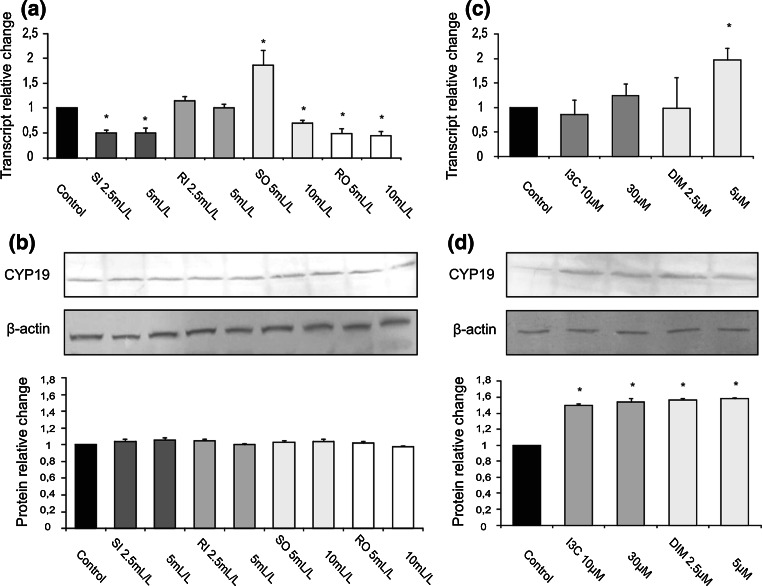
The effect of 72 h incubation with cabbage juices (**a**, **b**) and indoles (**c**, **d**) on the level of the *CYP19* transcript (**a**, **c**) and protein (**b**, **d**) in MDA-MB-231 cells. The values were calculated as a relative change in transcript or protein level in comparison with control cells (expression equals 1). The mean values ± SEM from three independent experiments run in duplicate (protein) or triplicate (mRNA) are presented. The *asterisk above the bar* denotes significant difference of mean values from the control group (*P* < 0.05)

In estrogen-independent MDA-MB-231 cells, cabbage juices depending on their origin and processing they either reduced, had no effect or increased *CYP19* transcript (Fig. 3a) and, similarly as in ER-positive MCF7 cells, cabbage juices did not change the aromatase protein level (Fig. 3b). DIM at the concentration of 5 μM increased *CYP19* transcript (2.0-fold change, Fig. 3c) in comparison with the control cells (treated with DMSO), while both indoles enhanced aromatase protein level (1.5–1.6-fold change, Fig. 3d).

**Fig. 4:**
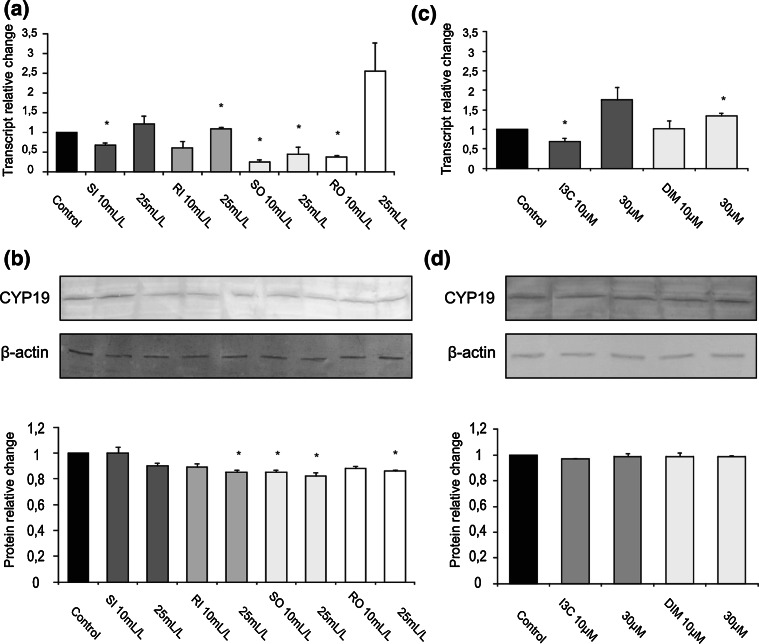
The effect of 72 h incubation with cabbage juices (**a**, **b**) and indoles (**c**, **d**) on the level of the *CYP19* transcript (**a**, **c**) and protein (**b**, **d**) in MCF10A cells. The values were calculated as a relative change in transcript or protein level in comparison with control cells (expression equals 1). The mean values ± SEM from three independent experiments run in duplicate (protein) or triplicate (mRNA) are presented. The *asterisk above the bar* denotes significant difference of mean values from the control group (*P* < 0.05)

The most interesting changes as result of cabbage juices treatment were observed in MCF10A cells. Most raw cabbage and sauerkraut juices significantly reduced the expression of *CYP19* at the level of both the transcript and the protein (0.25–0.82-fold change, Fig. 4a, b).

Surprisingly, RI in a dose of 25 mL/L increased mRNA (Fig. 4a), but decreased its protein level (Fig. 4b). In this cell line, I3C (10 μM) decreased *CYP19* transcript (0.68-fold change, Fig. 4c), while DIM (10 μM) increased it (1.3-fold change, Fig. 4c), but they both did not affect its protein level (Fig. 4d).

Interestingly, in general, the decrease in aromatase expression was more exerted by lower doses of tested juices and indoles (Figs. 2c, d; 3a; 4a).

### Effect of cabbage juices and indoles on apoptosis induction

**Fig. 5:**
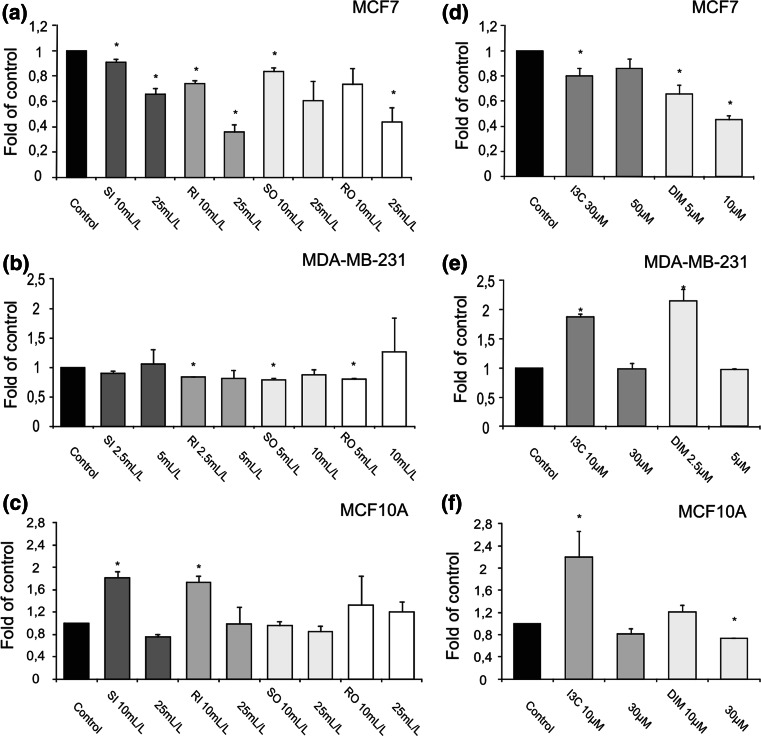
The effect of cabbage juices and indoles on apoptosis in MCF7 (**a**, **d**), MDA-MB-231 (**b**, **e**), and MCF10A (**c**, **f**) cells. The mean values ± SEM from three independent cell culture experiments run in triplicate are shown. The *asterisk above the bar* denotes statistically significant differences from the control group (*P* < 0.05)

The results of ELISA test detecting histone/DNA fragments are shown in Fig. 5. Juices obtained from cabbage grown on industrial farm-induced apoptosis in MCF10A cells, but only in lower doses (1.7–1.8-fold change, Fig. 5c), while in breast cancer cells particularly MCF7 the anti-apoptotic effect was observed as result of juices’ treatment (0.36–0.91-fold change) except SO in 25 mL/L and RO in 10 mL/L (Fig. 5a). In lower concentrations, I3C and DIM-induced apoptosis in ER-negative MDA-MB-231 (1.9–2.2-fold change, Fig. 5e), but only I3C in a dose 10 μM shown such activity in nontumorigenic MCF10A cells (2.2-fold change, Fig. 5f). In ER-positive cancer cells, treatment with these compounds (except 50 μM I3C) resulted in reduced level of histone/DNA fragments in comparison with control untreated cells (0.45–0.80-fold change, Fig. 5d). Similarly, in MCF10A cells, only DIM in dose 10 μM exerted anti-apoptotic effect (0.73-fold change, Fig. 5f). Moreover, as in expression analyses, the proapoptotic effect of tested juices and indoles was usually more evident in lower doses.

## Discussion

Estrogens are a key regulator of the proliferation and differentiation of breast cancer cells. In addition to the estrogen supply from the ovary, estrogens are produced locally from androgen by aromatase. Although AIs are important drugs in hormonal therapy for breast cancer in postmenopausal women, there are concerns about the side effects associated with the estrogen deprivation achieved with AIs [20]. Phytochemicals are considered attractive alternative to synthetic drugs, and many have been documented to be aromatase inhibitors in vitro and/or in vivo [9]. They represent different classes of natural products, but except red wine extracts [8], none of the studies on the potential aromatase inhibitors included the natural food matrix.

In our study, we evaluated the effect of cabbage and sauerkraut juices as well as their major ingredients—indoles (I3C and DIM) on aromatase expression in three breast cell lines. MCF7 cell line is the most widely studied breast carcinoma cell line because of its steroid receptor status and estrogen sensitivity [21]. In contrast to MCF7 cells, MDA-MB-231 cells are ER-negative and have aggressive invasion capacity [22]. The parental MCF10A cell line was derived from spontaneously immortalized breast epithelial cells [22] and is frequently used as a normal control in breast cancer studies [23].

Our real-time PCR analysis revealed the expression of *CYP19* in all cell lines tested, but despite the differences in their characteristics the levels of *CYP19* transcript did not differ substantially. While the presence of aromatase transcripts and protein in MCF7 cells was described by several authors [21, 24, 25], the data on constitutive expression of *CYP19* in this cell line as well as in MCF10A are somehow contradictory. In this regard, Fu et al. [22] did not find the presence of *CYP19* mRNA in MCF10A series of cell lines or MCF7 cells. However, they found the trichostatin A (histone deacetylase inhibitor) inducible effect on *CYP19* expression in all of the cells examined.

In our study, the presence of *CYP19* transcript was accompanied by detectable level of protein as measured by Western blot further supporting the presence of aromatase in all tested cell lines. Remarkable differences in the effect on the tested cell lines were found between the cabbage juices and pure indoles. Based on MTT assay, the doses of cabbage juices and indoles applied were consistently >70 % in all cell lines. However, in case of cabbage juices, differences in the cytotoxic effects between the cell lines were observed with MDA-MB-231 being the most sensitive and MCF10A the least sensitive. No such difference was found in case of indoles.

Moreover, the sauerkraut and some raw cabbage juices mostly affected the aromatase expression in nontumorigenic immortalized MCF10A breast cells, while the indoles, I3C and DIM were more efficient in decreasing the aromatase expression in estrogen-dependant MCF7 breast cancer cells. Among the juices, sauerkraut juice obtained from cabbage grown on organic farm was the most potent inhibitor of *CYP19* expression both on mRNA and protein level in MCF10A cells. Our previous study showed that sauerkraut juice was also more efficient than raw cabbage juice in induction of phase II enzymes in vivo in rats [19]. The sauerkraut is the product of lactic acid fermentation of shredded and salted white cabbage. During shredding, glucobrassicin, the most commonly studied GLS, is transformed into I3C by the action of myrosinase. During fermentation, as the pH decreases, this I3C reacts nonenzymatically with L-ascorbic acid to yield ABG, which is thought to be the dominant end product of indole GLS degradation in sauerkraut [12, 14]. So far only a few and rather indirect studies on the anti-carcinogenic effects of ABG have been published, and its interaction with aryl hydrocarbon receptor (AhR) was suggested [24]. Thus, it is possible that ABG is also responsible for down-regulation of *CYP19* expression by sauerkraut juice in MCF10A cell line.

Inhibition of aromatase by cabbage juices, particularly by SI in a concentration 10 mL/L, was correlated with the induction of apoptosis in MCF10A cells, which indirectly indicates that suppression of in situ estrogen formation by this cabbage juice may result in programmed cell death. Although MCF10A cells have normal mammary epithelial cell morphology, at the same time they possess a characteristic that make them a good model of breast tumor promotion [23]. This stage of carcinogenesis is characterized by clonal expansion of the initiated cells and is the most desired target of chemoprevention strategy [26]. Thus, the data provided in this study support epidemiological observations and justified application of white cabbage products in breast cancer early prophylaxis. In MDA-MB-231, ER-negative cancer cells, cabbage juices increased or decreased level of *CYP19* mRNA depending on dose. This opposite effect was seen particularly in the case of sauerkraut juice obtained from cabbage grown on organic farm was observed. Up-regulation of aromatase expression was also found in MCF7 cells as result of treatment with the others cabbage juices. In both cell lines, this effect was observed on mRNA level, while protein level was not changed. While the opposite effect of different doses can be explained in part by hormetic effect [27], the discrepancy between the effect on mRNA and protein level requires further studies. It is also worth noting that cauliflower juice studied in the same breast cancer cell lines exerted cytostatic (anti-proliferative) or cytotoxic effect depending on juice concentration and cell line [28].

I3C is a phytochemical that has been documented in numerous epidemiological and preclinical studies to posses mammary cancer preventive properties [29]. Once I3C reaches the acidity of the stomach, it can be converted to derivatives such as DIM. In cultured breast cancer cells, I3C has been shown to be anti-proliferative and apoptotic agent [15, 16]. It was suggested that anti-tumorigenic effects of I3C in MCF7 human breast cancer cells may arise from its ability to reduce ERα expression through the binding of its metabolite, DIM to nuclear AhR [30].

Although our study did not confirm the proapoptotic activity of both compounds in MCF7 cell line, provided the first evidence that they may also down-regulate the expression of *CYP19*. Moreover, DIM exerted this activity in a dose of 5 μM, which was 6 times lower than that of I3C. It has to be stressed that in this study I3C and DIM in the dose (100 μM) used by Wang et al. [30] showed significant cytotoxicity. The lower doses used were equivalent to those achieved in plasma [31].

Several studies on MDA-MB-231 and MCF10A cell lines have shown proapoptotic activity of both indoles [31–33] and DIM [34–36]. Results of this study confirmed proapoptotic activity of I3C in a dose 10 μM in ER-negative MDA-MB-231 and nontumorigenic MCF10A cells, but not in ER-positive MCF7 cell line. We have also demonstrated that DIM in a dose 5 μM could favor apoptosis of MDA-MB-231, but not MCF10A and MCF7 cells, where anti-apoptotic effect was observed.

Moreover, in most cases, lower doses of juices and indoles seem to be more efficient in proapoptotic effects and in decreasing aromatase expression, and conversely less efficient in anti-apoptotic effects and in induction of aromatase expression. This observation could be partially explained by hormetic phenomena mentioned above [27].

In summary, it must be stressed that the interpretation of some results presented in this paper could be seen as somehow contradictory when changes in RNA level are not reflected in protein concentration. Thus, more studies are required in order to confirm the results of our current study. These will include a more sensitive protein measurement technique and several cell lines differing in tumorigenicity/invasiveness.

Nevertheless, we suggests that the results of this study indicating down-regulation of *CYP19* mRNA and protein expression by cabbage juices observed in breast nontumorigenic MCF10A cell line may explain the chemopreventive activity of white cabbage and sauerkraut observed in epidemiological studies. Natural product research has continued to supply potent aromatase inhibitory lead compounds that provide interesting new avenues of investigation. White cabbage and its active components may be one of them.

Up-regulation of *CYP19* expression by I3C and DIM, and together with pro-apoptotic activity observed in ER-negative MDA-MB-231 cells, suggests that inhibitors of aromatase expression may be applied only for chemoprevention and/or therapy of ER-positive breast tumors and, as shown in a recent study of Murillo et al. [37], multifunctional signal transduction inhibitors such as deguelin should be sought in order to prevent ER-negative breast cancer.
